# Swimmer’s Lung?: Indoor Pools and Respiratory Effects

**DOI:** 10.1289/ehp.112-a1010a

**Published:** 2004-12

**Authors:** Angela Spivey

Bronchiolar epithelium cells known as Clara cells are thought to help defend airways against damage; Clara cell protein (CC16) is a lung-specific protein thought to protect the respiratory tract from inflammation. Studies have shown a positive association between ozone exposure and increased concentrations of CC16 in blood serum, leading researchers to suggest that ozone exposure damages the lung epithelium, causing it to “leak” proteins such as CC16. Now Birgitta Json Lagerkvist of Umeå University in Sweden and colleagues present findings that complicate that idea **[*EHP* 112:1768–1771]**.

Lagerkvist and colleagues report that children who regularly visited indoor swimming pools actually showed decreased concentrations of CC16 both before and after sessions of exercise outdoors in ambient levels of ozone. The researchers theorize that repeated exposure to disinfection by-products around indoor swimming pools may damage Clara cells, decreasing production of CC16 and possibly masking any effect that ozone exerts on CC16 levels.

This report is part of a set of studies examining possible changes in CC16 serum levels in relation to ambient ozone exposure as well as exposure to other environmental agents such as the chlorine around swimming pools and its by-products. In addition to the current study, which examined children in the summer, three other studies have examined children in winter, adults in summer, and adults in winter.

Subjects were screened using blood samples, lung function tests, and questionnaires. The final 57 subjects were 33 boys and 24 girls aged 10–11 years who had no asthma, pollen allergy, or initial decreased lung function. One subset of the 34 children had visited an indoor pool for at least one hour per month for six months or longer; the remaining 23 children had not.

The children exercised lightly outdoors for two hours on the Umeå University campus, where the ambient ozone was a moderate 77–116 micrograms per cubic meter. Lung function testing and blood sampling were conducted both before and after exposure.

The researchers saw no impairment of lung function in any of the children, and in looking at the entire study group, they did not find any statistically significant relationships between ozone exposure and CC16 concentrations. Among the children who did not visit swimming pools, there was a marginally significant tendency toward such a correlation. However, the children who regularly visited chlorinated indoor swimming pools showed significantly lower CC16 serum concentrations both before and after ozone exposure, compared to those who didn’t visit pools.

The authors say this may indicate that repeated exposure to disinfection by-products around indoor swimming pools damages Clara cell function. This theory is supported by previous studies, including one by report coauthor Alfred Bernard that showed an association between regular pool attendance among people taking swimming lessons and reduced CC16 concentrations. The researchers speculate that if regular indoor pool attendance does decrease CC16 production, then this effect may mask any increased leakage of CC16 caused by ozone. They further suggest that such masking may have happened in this study, indicated by the slight tendency to show a correlation between ozone exposure and increased CC16 concentrations only in the children who did not visit swimming pools.

They conclude that Clara cell damage associated with indoor pool use may diminish the anti-inflammatory effect of CC16 in the lung. Another previous study by Bernard showed increased incidence of asthma among children who regularly visited indoor pools, and the authors of the current report call for further investigation of the possibility that exposure to disinfection byproducts around indoor pools can play a role in inducing asthma through impaired Clara cell function.

## Figures and Tables

**Figure:**
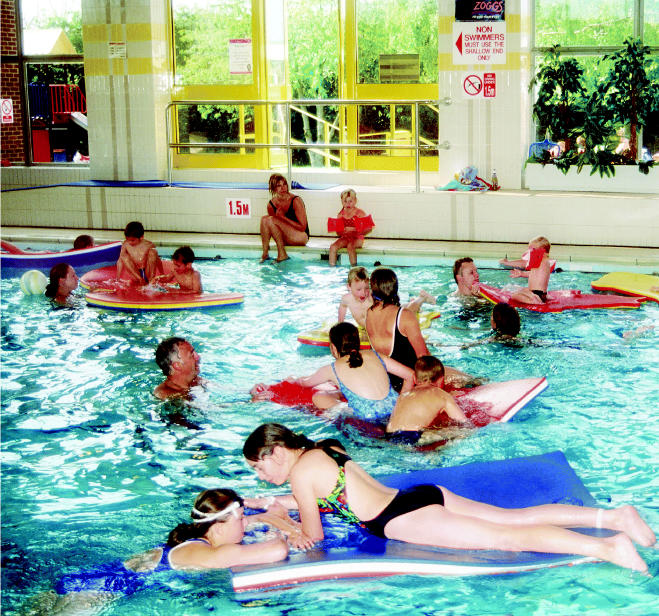
Bathers beware? Repeated exposure to disinfection by-products around indoor swimming pools may damage cells that produce protective lung-specific proteins.

